# Tuberculose no sistema prisional brasileiro: cenários via Joinpoint entre 2007 e 2019 

**DOI:** 10.1590/0102-311XPT166722

**Published:** 2023-09-25

**Authors:** Nancy Meriane de Nóvoa-Lôbo, Mônica Rodrigues Campos, Débora Castanheira Pires

**Affiliations:** 1 Escola Nacional de Saúde Pública Sergio Arouca, Fundação Oswaldo Cruz, Rio de janeiro, Brasil.; 2 Instituto de Comunicação e Informação Científica e Tecnológica em Saúde, Fundação Oswaldo Cruz, Rio de Janeiro, Brasil.

**Keywords:** Prisões, Prisioneiros, Tuberculose, Análise de Regressão, Prisons, Prisoners, Tuberculosis, Regression Analyses, Prisiones, Prisioneros, Tuberculosis, Análisis de Regresión

## Abstract

Este estudo descreveu e comparou dados de tuberculose (TB) entre pessoas privadas de liberdade e população geral brasileira, de 2007 a 2019, utilizando a ferramenta Joinpoint para observação de mudanças de tendências. Apresenta um recorte para mulheres e idosos, para testagem para HIV e para número de custodiados por vaga. Trata-se de um estudo retrospectivo, quantitativo e analítico, que utiliza métodos de regressão de dados de séries temporais a partir de dados secundários de acesso irrestrito coletados do Sistema de Informação de Agravos de Notificação (SINAN), do Instituto Brasileiro de Geografia e Estatística (IBGE) e de relatórios analíticos disponibilizados pelo Departamento Penitenciário Nacional (DEPEN). Os resultados retratam aumento da prevalência de TB consideravelmente maior em pessoas privadas de liberdade em todas as perspectivas analisadas; aumento nas testagens para HIV; e discutível tendência de estabilidade na quantidade de custodiados por vaga. Ao se analisar tendências de prevalências, serviços e determinantes, é curioso ver a não coincidência temporal na maioria dos casos. Ficou claro que as políticas nacionais de combate à TB não têm o mesmo efeito dentro das prisões e mesmo a Política Nacional de Atenção Integral à Saúde das Pessoas Privadas de Liberdade no Sistema Prisional (PNAISP) mostrou efeitos restritos diante da situação de saúde aqui analisada. Apesar de trabalhar com dados secundários de confiabilidade variável, alcançaram-se comparações que podem impactar decisões e ações de saúde. Ainda que carente de respostas completas e definitivas, pôde-se lançar um novo olhar à evolução de questões sobre as quais a reflexão é imprescindível.

## Abstract (en)

This study describes and compares tuberculosis (TB) data among persons deprived of liberty and the general Brazilian population, from 2007 to 2019, using the Joinpoint tool to observe changes in trends. This study focuses on women and older adults, for HIV testing, and on the number of detainees according to prison capacity. This is a retrospective, quantitative, and analytical study, which uses methods of regression of time series data from secondary data of unrestricted access collected from the Brazilian Information System for Notifiable Diseases (SINAN), Brazilian Institute of Geography and Statistics (IBGE), and from analytical reports made available by the Brazilian National Penitentiary Department (DEPEN). The results show a considerably higher increase in the prevalence of TB in persons deprived of liberty in all perspectives analyzed; increased HIV testing; and a debatable trend of stability in the number of detainees according to prison capacity. When analyzing trends in prevalence, services, and determinants, it is curious to see the temporal non-coincidence in most cases. Clearly, national policies against TB do not have the same effect within prisons and even the National Policy for Comprehensive Health Care for People Deprived of Liberty in the Prison System (PNAISP) showed restricted effects in view of the health situation herein analyzed. Despite working with secondary data of variable reliability, comparisons were reached that can impact health decisions and actions. Although lacking complete and definitive answers, it was possible to launch a new point-of-view on the evolution of questions for which reflection is essential.

## Resumen

El presente estudio describió y comparó los datos de tuberculosis (TB) entre las personas privadas de libertad y la población general brasileña, de 2007 a 2019, utilizando la herramienta Joinpoint para observar los cambios en las tendencias. Presenta un límite para mujeres y ancianos, para las pruebas para VIH y para el número de internos por vacante. Se trata de un estudio retrospectivo, cuantitativo y analítico, que utiliza métodos de regresión de datos de series temporales a partir de datos secundarios de acceso no restringido recolectados del Sistema de Información de Agravios de Notificación (SINAN), Instituto Brasileño de Geografía y Estadística (IBGE) e informes analíticos disponibles por el Departamento Penitenciario Nacional (DEPEN). Los resultados muestran un aumento de la prevalencia de TB considerablemente mayor en personas privadas de libertad en todas las perspectivas analizadas; un aumento en las pruebas de VIH, y una tendencia discutible a la estabilidad en la cantidad de internos por vacante. Al analizar las tendencias de las prevalencias, los servicios y determinantes, es curioso ver la no coincidencia temporal en la mayoría de los casos. Quedó claro que las políticas nacionales de combate a la TB no tienen el mismo efecto dentro de las prisiones y incluso la Política Nacional de Atención Integral a la Salud de las Personas Privadas de Libertad en el Sistema Penitenciario (PNAISP) mostró efectos restringidos frente a la situación de salud aquí analizada. A pesar de trabajar con datos secundarios de confiabilidad variable, se lograron comparaciones que pueden afectar las decisiones y acciones de salud. Aunque se carezca de respuestas completas y definitivas, se ha podido dar una nueva mirada a la evolución de cuestiones sobre los que es imprescindible reflexionar.

## Introdução

A tuberculose (TB) atinge a humanidade há milênios ^1^. É uma doença infectocontagiosa e conhecer seu perfil epidemiológico é fundamental para reduzir o tempo entre os primeiros sintomas, o diagnóstico e o início do tratamento medicamentoso supervisionado ^2^. Falhas na organização dos sistemas de atenção primária à saúde levam a diagnósticos tardios, resultando em taxas mais altas de mortalidade ^2^.

Sabe-se que pessoas privadas de liberdade estão entre as mais vulneráveis à doença ^3^^,^^4^. Globalmente, pessoas privadas de liberdade são mais afetadas por doenças infecciosas do que a população geral não presa ^5^ e é incontestável o agravamento de condições patológicas previsíveis em situação de confinamento. Em que pese não haver descrições de indicadores dos boletins epidemiológicos nacionais da TB específicos para a população prisional, assume-se que a TB tem uma incidência até 35 vezes maior em pessoas privadas de liberdade quando comparada à população geral ^6^.

A conhecida superlotação dos presídios brasileiros, por exemplo, associada a uma estrutura física insalubre, é fator determinante para deterioração da saúde no contexto prisional, principalmente para doenças respiratórias como TB e, mais recentemente, infecções pelo coronavírus ^7^^,^^8^^,^^9^. Além disso, os indicadores relacionados à TB em pessoas privadas de liberdade retratam uma situação de acesso aos serviços de saúde mais precária do que a que acontece extramuros ^10^.

Diante da necessidade de melhoria das condições de saúde de pessoas privadas de liberdade, o Plano Nacional de Saúde no Sistema Penitenciário (PNSSP) foi a primeira normativa brasileira a sistematizar a assistência à saúde para presos ^11^. Já em 2014, foi promulgada a Política Nacional de Atenção Integral à Saúde das Pessoas Privadas de Liberdade no Sistema Prisional (PNAISP), corrigindo algumas lacunas do plano anterior ^12^. Ainda que não haja dúvidas sobre a importância da PNAISP na evolução do cuidado em saúde prisional, sua formulação careceu de pesquisa epidemiológica abrangente ^13^.

Embora o número de pesquisas sobre saúde no sistema prisional tenha aumentado a partir da década de 1990 nos cinco continentes ^14^, grande parte dos estudos disponíveis são do tipo transversal e não capturam a dinâmica da doença nessa população ^15^ devido a alguns aspectos, tais como: comprometimento do nível de abrangência da unidade de análise, ou seja, estudam unidades prisionais específicas ^16^^,^^17^ ou uma única Unidade Federativa (UF) ^18^; restrições quanto ao nível de comparabilidade dos indicadores de desfecho no tempo, por exemplo, abordam análises temporais para pessoas privadas de liberdade em comparação a dados não desagregados por fatores determinantes para a população geral ^10^.

De modo a suprir tais lacunas, este estudo pretende descrever e comparar dados de TB entre pessoas privadas de liberdade e população geral brasileira, de 2007 a 2019, utilizando a ferramenta Joinpoint para observação de mudanças de tendências ao longo da série temporal.

## Metodologia

### Fontes do estudo

Trata-se de um estudo retrospectivo, quantitativo e analítico, que utiliza métodos de regressão de dados de séries temporais. Os dados relativos à TB foram obtidos por meio do Sistema de Informação de Agravos de Notificação (SINAN). Para descrição da população geral, os dados foram colhidos do Instituto Brasileiro de Geografia e Estatística (IBGE), ambos mediante ferramenta Tabnet. As informações relativas às pessoas privadas de liberdade, bem como à infraestrutura prisional, foram obtidas através dos relatórios analíticos disponibilizados pelo Departamento Penitenciário Nacional (DEPEN). Ressalta-se que, neste trabalho, o termo pessoas privadas de liberdade não engloba pessoas acolhidas em carceragens de delegacias e congêneres não administradas pelas secretarias de administração penitenciária.

Sinaliza-se a problemática existente quanto à mudança de variáveis nas fichas de notificação do SINAN, em 2014, para os dados de TB, quando excluiu-se o termo institucionalizado em presídios e incluiu-se pessoas privadas de liberdade. Além disso, constata-se baixa completude de campos relacionados a populações especiais, bem como a não atualização de testes para HIV ^19^. Apesar disso, a TB é a única doença cujas pessoas privadas de liberdade são identificadas no SINAN e apresenta uma série temporal apreciável.

Os relatórios do DEPEN são construídos pelo Sistema de Informações Estatísticas do Sistema Penitenciário Brasileiro (Infopen) ^20^. Apesar de várias limitações de informações importantes, além de a mudança da apresentação dos modelos de relatórios dificultar a construção de uma série temporal, seus relatórios sintéticos e analíticos são os únicos documentos que descrevem o total da população prisional brasileira.

Apesar de a disponibilização de relatórios pelo DEPEN acontecer desde 2005, o recorte temporal deste estudo restringe-se a 2007-2019 devido à baixa completude de dados do SINAN em 2005 e 2006, com apenas 5% e 22,2% das notificações cujo campo institucionalizado estava preenchido.

### Variáveis de análise

Os indicadores foram calculados a partir dos dados brutos das fontes descritas acima com vistas à apresentação de resultados compatíveis com o que usualmente se encontra na literatura, facilitando a comparabilidade.

As variáveis criadas estão explicadas baixo:

(1) Prevalência de TB em pessoas privadas de liberdade (PREV TB TOT PPL): casos confirmados de TB em presídio ou pessoas privadas de liberdade (SINAN)/Total pessoas privadas de liberdade (DEPEN);

(2) Prevalência de TB na população geral brasileira (PREV TB BR): casos confirmados de TB no Brasil (SINAN)/Total população brasileira (IBGE);

(3) Prevalência de TB em mulheres privadas de liberdade (PREV TB PPL FEM): casos confirmados de TB em presídio ou pessoas privadas de liberdade do sexo feminino (SINAN)/Total pessoas privadas de liberdade do sexo feminino (DEPEN)

(4) Prevalência de TB na população geral feminina (PREV TB POP FEM): casos confirmados de TB no Brasil do sexo feminino (SINAN)/Total população brasileira do sexo feminino (IBGE);

(5) Prevalência de TB em pessoas privadas de liberdade com 60 ou mais anos (PREV TB PPL 60+): casos confirmados de TB em presídio ou pessoas privadas de liberdade com 60ou mais anos (SINAN)/Total pessoas privadas de liberdade com 60 ou mais anos (DEPEN);

(6) Prevalência de TB na população geral idosa com 60 ou mais anos (PREV TB BR 60+): casos confirmados de TB no Brasil em pessoas com 60 ou mais anos (SINAN)/Total população brasileira com 60 ou mais anos (IBGE);

(7) Razões entre as prevalências de TB em pessoas privadas de liberdade e população geral (RAZÃO PREV TB PPL/POP): prevalência de TB em pessoas privadas de liberdade (SINAN)/Prevalência de TB na população geral (SINAN);

(8) Razões entre as prevalências de TB em pessoas privadas de liberdade mulheres e população geral feminina (RAZÃO PREV TB PPL FEM/POP FEM): prevalência de TB em pessoas privadas de liberdade feminina (SINAN)/Prevalência na população geral feminina (SINAN);

(9) Razões entre as prevalências de TB em pessoas privadas de liberdade com 60ou mais anos e população geral idosa (RAZÃO PREV TB PPL 60+/POP 60+): prevalência de TB em pessoas privada de liberdade com 60 ou mais anos (SINAN)/Prevalência de TB na população geral com 60 ou mais anos (SINAN);

(10) Porcentagem de pessoas privadas de liberdade diagnosticada com TB testada para HIV (% TESTE HIV): casos de TB testados para HIV em presídios ou pessoas privadas de liberdade (SINAN) × 100/Casos confirmados de TB em presídio ou pessoas privadas de liberdade (SINAN);

(11) Número de pessoas privadas de liberdade por vaga (PPL/VAGA): total pessoas privadas de liberdade (DEPEN)/Total de vagas (DEPEN).

### Análise estatística dos dados

Para análise estatística e construção de gráficos de tendência temporal, empregou-se o software Joinpoint Regression Program, versão 4.9.1.0 (https://surveillance.cancer.gov/joinpoint/), que modela a série utilizando um método de regressão ponto a ponto, em que a variação é estimada através da regressão de Poisson e os testes de significância para a mudança de tendência utilizam o método de permutação de Monte Carlo. Os dados deste estudo foram analisados sob o pressuposto de variância constante (homocedasticidade) e autocorrelação de primeira ordem.

O programa de regressão Joinpoint é um software de análise de tendências desenvolvido pelo Instituto Nacional do Câncer dos Estados Unidos (NCI) ^21^. O objetivo desse programa é evidenciar os pontos de junção, ou “joinpoints”, em que há mudança na tendência evolutiva da variável considerada ^22^. Assim sendo, no modelo final, cada ponto de junção informa uma mudança estatisticamente significativa nas tendências (aumento ou diminuição) e cada uma dessas tendências é descrita por um coeficiente (*slope*) ^21^. No modelo, cada ponto destacado de inflexão da tendência é significativo (valor de p < 5%), mesmo que o *slope* de uma das segmentações não o seja.

### Aspectos éticos

Por utilizar apenas dados secundários de acesso livre e irrestrito, não houve necessidade de submissão desta pesquisa ao Comitê de Ética, de acordo com a *Resolução nº 510*, de 7 de abril de 2016 ^23^, do Conselho Nacional de Saúde.

## Resultados

O número de pessoas privadas de liberdade custodiadas pelo sistema prisional brasileiro cresceu 293,8% de 2007 a 2019. Da mesma forma, aumentou a quantidade de pessoas custodiadas pelo sistema prisional diante da população geral (de 193,4 para 355,1), incluindo a população carcerária feminina (19,9 para 34,6), considerando o total de mulheres no Brasil, e a população carcerária de idosos de 60 anos ou mais, tendo em vista a população brasileira da mesma idade (19,1 para 36,5) (Tabela 1).

**Tabela 1:** Caracterização da população prisional geral, feminina e de 60 ou mais anos e número de pessoas privadas de liberdade por vaga. Brasil, 2007-2019.

Ano	PPL BR	TAXA PPL	% PPL FEM	TAXA PPL FEM	% PPL 60+	TAXA PPL 60+	PPL/VAGA
2007	366.359	193,4	5,2	19,9	0,9	19,1	1,5
2008	394.488	206,0	5,5	22,3	0,8	18,3	1,5
2009	417.112	215,5	5,8	24,8	1,0	21,6	1,5
2010	445.705	228,0	6,3	28,5	1,0	22,2	1,6
2011	471.254	238,7	6,2	29,4	1,0	23,8	1,6
2012	548.003	275,0	5,8	31,4	0,9	23,8	1,8
2013	537.790	267,5	6,1	32,1	1,0	24,2	1,7
2014	584.758	288,4	5,8	32,9	1,0	24,5	1,6
2015	663.385	324,5	5,6	36,1	0,8	22,9	1,8
2016	702.385	340,8	5,7	38,1	1,0	27,7	1,6
2017	704.576	339,3	5,3	35,7	1,1	30,2	1,6
2018	725.332	346,7	4,9	33,3	1,1	30,0	1,6
2019	748.009	355,1	4,9	34,6	1,4	36,5	1,7

% PPL 60+: porcentagem de pessoas privadas de liberdade de 60 ou mais anos no sistema prisional brasileiro; % PPL FEM: porcentagem de pessoas privadas de liberdade mulheres no sistema prisional brasileiro; PPL BR: total de pessoas privadas de liberdade custodiadas pelos sistemas prisionais estaduais brasileiros; PPL/VAGA: número de pessoas privadas de liberdade por vaga no sistemas prisionais estaduais brasileiro; TAXA PPL: razão do número de pessoas privadas de liberdade por 100 mil habitantes; TAXA PPL 60+: número de pessoas privadas de liberdade com 60 ou mais anos por 100 mil habitantes brasileiros da mesma idade; TAXA PPL FEM: número de pessoas privadas de liberdade mulheres por 100 mil mulheres brasileiras.

Fonte: população do Brasil: Instituto Brasileiro de Geografia e Estatística ^53^; pessoas privadas de liberdade: relatórios analíticos do Departamento Penitenciário Nacional (DEPEN) ^20^; dados sobre tuberculose: Sistema de Informação de Agravos e Notificações (SINAN) ^19^.

No mesmo período, o percentual de mulheres privadas de liberdade praticamente permanece estável (5,2% para 4,9%) enquanto o de idosos privados de liberdade de ambos os gêneros se tornou maior diante do total da população penitenciária. O número de pessoas por vaga disponível nos presídios e congêneres brasileiros também foi maior ao final da série temporal (1,7 pessoas privadas de liberdade por vaga) (Tabela 1).

A descrição da prevalência de TB para pessoas privadas de liberdade e população geral, feminina e de 60 anos ou mais, e as razões entre esses públicos estão expostas na Tabela 2. Todas as prevalências de TB são largamente superiores em pessoas privadas de liberdade quando comparadas à população geral. As prevalências entre esses públicos chegaram a ser 38,8 vezes maiores em privados de liberdade comparados à população geral em 2011; 69,9 vezes maiores para pessoas privadas de liberdade feminina e 83,0 vezes maiores para pessoas privadas de liberdade com 60 ou mais anos, ambos em 2008. Não há, entretanto, fluxo contínuo de aumento ou diminuição das taxas e, consequentemente, das razões. Porém, considerando os extremos do período, observam-se aumento da razão pessoas privadas de liberdade e população geral de 22 para 34 e redução da razão entre sexo feminino (69,7 a 30,8) e idosos (79,1 a 30,8).

**Tabela 2:** Descrição da prevalência de tuberculose (TB) em pessoas privada de liberdade e na população geral, feminina e de 60 ou mais anos. Brasil, 2007-2019.

Ano	PREV TB TOT PPL	PREV TB BR	RAZÃO PREV TB PPL/POP	PREV TB PPL FEM	PREV TB POP FEM	RAZÃO PREV TB PPL FEM/POP FEM	PREV TB PPL 60+	PREV TB BR 60+	RAZÃO PREV TB PPL 60+/POP 60+	% TESTE HIV
2007	983	44,7	22,0	2.085,7	29,9	69,7	4.874,4	61,6	79,1	56,8
2008	1.153	45,3	25,5	2.106,1	30,1	69,9	4.927,9	59,4	83,0	61,5
2009	1.351	44,6	30,3	1.901,9	29,5	64,4	3.925,4	57,2	68,6	66,2
2010	1.334	43,7	30,5	1.749,0	28,5	61,4	3.753,2	56,2	66,8	68,9
2011	1.726	44,5	38,8	1.884,3	28,7	65,6	4.186,4	57,0	73,4	75,0
2012	1.599	43,3	37,0	1.792,0	27,7	64,7	3.904,9	53,1	73,5	75,7
2013	1.343	42,9	31,3	1.641,3	27,5	59,8	3.506,5	52,1	67,3	77,0
2014	1.351	42,0	32,1	1.683,8	26,4	63,7	3.617,0	50,3	71,9	76,5
2015	1.154	41,8	27,6	914,9	25,5	35,8	2.442,1	49,8	49,0	83,3
2016	1.217	41,8	29,1	754,7	25,4	29,7	1.682,4	49,6	33,9	79,6
2017	1.456	43,6	33,4	719,2	25,7	28,0	1.962,3	50,6	38,8	80,4
2018	1.502	45,1	33,3	795,4	26,8	29,7	2.062,0	49,6	41,6	78,7
2019	1.558	45,5	34,2	828,6	26,9	30,8	1.538,0	49,9	30,8	80,5

% TESTE HIV: porcentagem de pessoas privadas de liberdade diagnosticadas com TB e testadas para HIV; PREV PPL 60+: prevalência de TB em pessoas privadas de liberdade com 60 ou mais anos; PREV TB BR: prevalência de TB na população geral brasileira; PREV TB TOT PPL: prevalência de casos confirmados de TB em pessoas privadas de liberdade; PREV TB POP FEM: prevalência de TB na população geral feminina; PREV TB PPL FEM: prevalência de TB em mulheres privadas de liberdade; PPL/VAGA: número de pessoas privadas de liberdade por vaga nos sistemas prisionais estaduais brasileiros; PREV TB BR 60+: prevalência de TB na população geral idosa com 60 ou mais anos; RAZÃO PREV TB PPL 60+/POP 60: razões entre as prevalências de TB em pessoas privadas de liberdade com 60 ou mais anos e população geral com 60 ou mais anos; RAZÃO PREV TB PPL FEM/POP FEM: razões entre as prevalências de TB em pessoas privadas de liberdade mulheres e população geral feminina; RAZÃO PREV TB PPL/POP: razões entre as prevalências de TB em pessoas privadas de liberdade e população geral.

Fonte: população do Brasil: Instituto Brasileiro de Geografia e Estatística ^53^; pessoas privadas de liberdade: relatórios analíticos do Departamento Penitenciário Nacional (DEPEN) ^20^; dados sobre tuberculose: Sistema de Informação de Agravos e Notificações (SINAN) ^19^.

Cabe destacar que, a despeito da acentuada relação entre o predomínio de pessoas privadas de liberdade diante da população geral, observa-se aumento da prevalência em pessoas privadas de liberdade no período, de 983 para 1558, enquanto houve expressiva redução da mesma entre mulheres (2.085,7 a 754,7) e pessoas custodiadas com 60 ou mais anos (4.874,4 a 1.962,3), bem como estabilidade dessa taxa para os mesmos grupos na população geral.

A proporção de custodiados diagnosticados com TB e testados para HIV também está na Tabela 2. Há aumento de testes executados, partindo de 56,8% em 2007 e atingindo 83,3% da população carcerária em 2015, com apenas três pontos de leve diminuição ao longo do período.

A Tabela 3 exibe os modelos Joinpoint para os indicadores apresentados na Tabela 2. A partir do modelo final, é possível afirmar que as mudanças de tendências nas prevalências de TB acontecem em momentos distintos quando comparadas pessoas privadas de liberdade e população geral. Por um lado, a prevalência de TB na pessoas privadas de liberdade cresce acentuadamente entre 2007 e 2011 (*slope* = 170,51), seguida de queda entre 2011 e 2015 (*slope* = -120,47), com posterior nova tendência de crescimento entre 2015 e 2019 (*slope* = 106,55). Por outro lado, a prevalência de TB na população geral tende a decair entre 2007 e 2016 (*slope* = -0,40), seguida de ligeira tendência de aumento entre 2016 e 2019 (*slope* = 1,49). Além disso, percebe-se que as razões de prevalência entre essas populações são determinadas pelo seu numerador, qual seja, pessoas privadas de liberdade. Assim, as mudanças de tendências das razões e de TB em pessoas privadas de liberdade são sincrônicas.

**Tabela 3:** Modelos de análise das mudanças de tendência nas séries temporais de prevalência de tuberculose (TB) para pessoas privadas de liberdade e população geral, feminina e de 60 ou mais anos, razões de prevalência, percentagem de teste de HIV e dados ausentes e razão de presos por vaga, utilizando modelos de regressão de Joinpoint. Brasil, 2007-2019.

	Dados 2007	Dados 2019	Tendência 1		Tendência 2		Tendência 3	
			Período	*Slope*	Período	*Slope*	Período	*Slope*
População geral								
PREV TB PPL	983,46	1.558,27	2007-2011	170.51 *	2011-2015	-120.47 *	2015-2019	106,55 *
PREV TB BR	44,65	45,55	2007-2016	-0,40 *	2016-2019	1,49 *		
RAZÃO PREV TB PPL/POP	22,02	34,21	2007-2011	4,02 *	2011-2015	-2,28	2015-2019	1,64 *
População feminina								
PREV TB PPL FEM	2.085,74	828,62	2007-2013	-52,60 *	2013-2016	-341,02	2016-2019	34,29
PREV TB POP FEM	29,92	26,93	2007-2016	-0,58 *	2016-2019	0,58 *		
RAZÃO TB PPL FEM/POP FEM	69,72	30,77	2007-2013	-0,60	2013-2016	-11,70	2016-2019	0,39
População com 60 anos ou mais								
PREV TB PPL 60+	4.874,40	1.538,01	2007-2019	-287,26 *				
PREV TB BR 60+	61,65	49,92	2007-2015	-1,44 *	2015-2019	0,16		
RAZÃO TB PPL 60+/POP 60+	79,07	30,81	2007-2019	-4,21 *				
Avaliação qualidade da atenção								
% TESTE HIV	56,84	80,50	2007-2011	4,18 *	2011-2015	1,66 *	2015-2019	-0,29
PPL/VAGA	1,50	1,70	2007-2012	0,05 *	2012-2019	-0,01 *		

% TESTE HIV: porcentagem de pessoas privadas de liberdade diagnosticadas com TB e testada para HIV; PPL/VAGA: número de pessoas privadas de liberdade por vaga nos sistemas prisionais estaduais brasileiros; PREV TB PPL 60+: prevalência de TB em pessoas privadas de liberdade de 60 ou mais anos; PREV TB BR 60+: prevalência de TB na população geral idosa de 60 ou mais anos; PREV TB BR: prevalência de TB na população geral brasileira; PREV TB POP FEM: prevalência de TB na população geral feminina; PREV TB PPL FEM: prevalência de TB em mulheres privadas de liberdade; PREV TB PPL: prevalência de casos confirmados de TB em pessoas privadas de liberdade; RAZÃO PREV TB PPL FEM/POP FEM: razão entre as prevalências de TB em pessoas privadas de liberdade feminina e população geral feminina; RAZÃO PREV TB PPL 60+/POP 60+: razões entre as prevalências de TB em pessoas privadas de liberdade com 60 ou mais anos e população geral com 60 ou mais anos; RAZÃO PREV TB PPL/POP: razões entre as prevalências de TB em pessoas privadas de liberdade e população geral.

Fonte: população do Brasil: Instituto Brasileiro de Geografia e Estatística ^53^; pessoas privadas de liberdade: relatórios analíticos do Departamento Penitenciário Nacional (DEPEN) ^20^; dados sobre tuberculose: Sistema de Informação de Agravos e Notificações (SINAN) ^19^.

* Tendências estatisticamente significativas.

A PREV TB PPL FEM tem evolução temporal bastante distinta da PREV TB PPL. A PREV TB PPL teve forte tendência de crescimento entre 2007 e 2011, só apresentando queda de 2011 a 2015. Em contrapartida, a PREV TB PPL FEM apresenta queda desde o início do período estudado (*slope* = -52,6), com subsequente intensificação da diminuição entre 2014 e 2016 (*slope* = -341,02) e posterior tênue tendência de crescimento entre 2016 e 2019 (*slope* = 34,29) (Tabela 3).

Enquanto isso, a PREV TB PPL FEM e a PREV TB POP FEM têm o mesmo padrão de evolução temporal, guardadas as diferenças de magnitude: queda entre 2007 e 2016, seguida de aumento entre 2016 e 2019, o que se reflete na similaridade da razão entre elas. Cabe destacar ainda que o padrão de tendências e inflexões para a prevalência na PREV TB BR e PREV TB POP FEM é o mesmo, o que mostra não existir diferentes perfis de adoecimento extramuros, considerando os sexos (Tabela 3).

A PREV TB PPL 60+ apresenta tendência temporal diferente de todas as demais descritas anteriormente, uma vez que tem contínua queda em todo o período (*slope* = -287,26). A PREV TB BR 60+ segue um padrão similar ao dos outros grupos de populações extramuros estudados (Tabela 3).

Ainda na Tabela 3, no que tange à avaliação da qualidade da atenção, quanto à porcentagem de teste HIV, encontramos uma tendência de crescimento entre 2007 e 2015, com acentuação em 2011 (*slope* = 4,18), seguida de leve queda de 2016 a 2019 (*slope* = -0,29). Para o indicador presos por vagas, observa-se ligeira tendência de crescimento de 2007 a 2012 (*slope* = 0,05), seguida de ínfima tendência de queda entre 2013 e 2019 (*slope* = -0,01).

**Figura 1:**
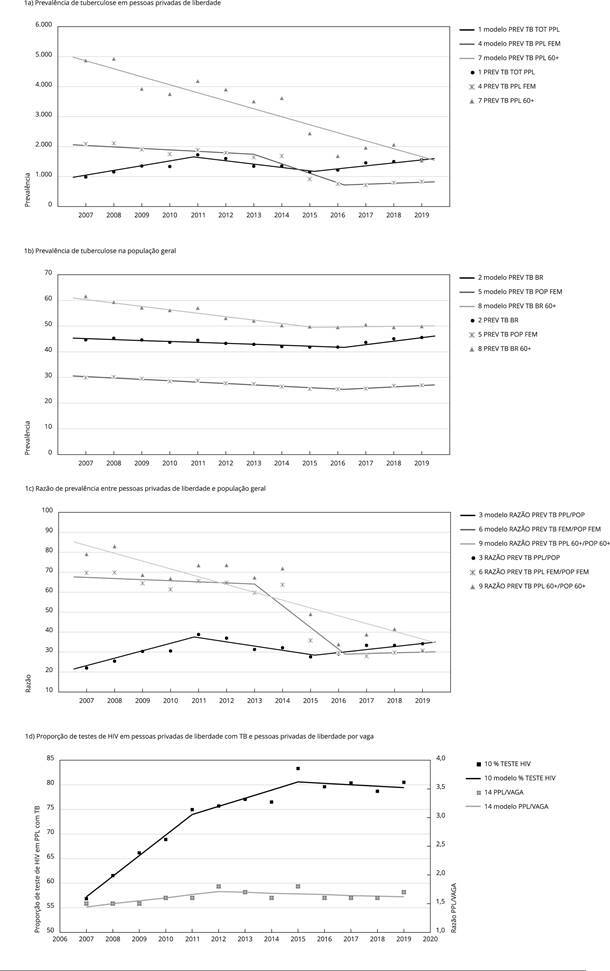
Séries temporais analisadas. Brasil, 2007-2019.

A Figura 1 apresenta a representação gráfica dos modelos de Joinpoint apresentados acima, para as prevalências de TB em pessoas privadas de liberdade (Figura 1a), prevalências de TB na população geral (Figura 1b), razão de prevalências entre essas populações (Figura 1c) e porcentagem de testagem de HIV para pessoas privadas de liberdade diagnosticadas com TB e número de presos por vaga (Figura 1d). A representação visual das séries permite perceber melhor as diferenças temporais relatadas anteriormente, tais como: a prevalência de TB muito superior na população privada de liberdade; as mudanças de razão de prevalência estabelecidas pelo adoecimento das pessoas custodiadas; um número de pessoas privadas de liberdade por vaga que, apesar de manter-se elevado, não apresenta grandes variações ao longo do tempo e, por isso, permanece um fator de risco constante para TB; e testagem de HIV com mudanças de tendências temporais semelhantes às da prevalência de TB em pessoas privadas de liberdade, embora no terceiro segmento da série o número de testes caia e a prevalência aumente.

% TESTE HIV: porcentagem de pessoas privadas de liberdade diagnosticada com TB e testadas para HIV; PPL/VAGA: número de pessoas privadas de liberdade por vaga nos sistemas prisionais estaduais brasileiros; PREV TB PPL 60+: prevalência de TB em pessoas privadas de liberdade com 60 ou mais anos; PREV TB BR 60+: prevalência de TB na população geral idosa com 60 ou mais anos; PREV TB BR: prevalência de TB na população geral brasileira; PREV TB POP FEM: prevalência de TB na população geral feminina; PREV TB PPL FEM: prevalência de TB em mulheres privadas de liberdade; PREV TB TOT PPL: prevalência de casos confirmados de TB em pessoas privadas de liberdade; RAZÃO PREV TB PPL FEM/POP FEM: razão entre as prevalências de TB em pessoas privadas de liberdade mulheres e população geral feminina; RAZÃO PREV TB PPL 60+/POP 60+: razões entre as prevalências de TB em pessoas privadas de liberdade com 60 ou mais anos e população geral com 60 ou mais anos; RAZÃO PREV TB PPL/POP: razões entre as prevalências de TB em pessoas privadas de liberdade e população geral. Fonte: população do Brasil: Instituto Brasileiro de Geografia e Estatística ^53^; pessoas privadas de liberdade: relatórios analíticos do Departamento Penitenciário Nacional (DEPEN) ^20^; dados sobre tuberculose: Sistema de Informação de Agravos e Notificações (SINAN) ^19^.

## Discussão

Este trabalho centra-se especialmente na comparação das prevalências de TB entre população prisional e população geral, apresentando também um recorte para mulheres e idosos e, ainda, um marcador de serviço - testagem para HIV - e outro de infraestrutura - número de custodiados por vaga. Os resultados aqui apresentados retratam o aumento da população prisional brasileira, tanto seus valores absolutos quanto aqueles relativos à população geral e ao número de vagas.

Constata-se ainda que a prevalência de TB em pessoas privadas de liberdade é consideravelmente maior que a da população geral em todas as perspectivas analisadas. Há um auspicioso aumento nas testagens para HIV. Os modelos Joinpoint e suas representações gráficas mostraram-se uma boa ferramenta para evidenciar as mudanças no período analisado.

O aumento da população carcerária brasileira condiz com a realidade mundial ^24^. Além disso, a crescente proporção de mulheres até 2013 também condiz com a literatura ^25^. Observa-se, porém, que de 2007 a 2019 a população privadas de liberdade cresceu 204,2%, enquanto para população privadas de liberdade feminina o aumento foi de 194%. Dessa forma, a estabilidade do percentual de mulheres custodiadas diante do total de pessoas privadas de liberdade no lapso temporal estudado ocorre por uma mudança não linear no percentual de mulheres.

O aumento da porcentagem de idosos custodiados, apesar de tímido (0,9 a 1,4%), é coincidente com a transição demográfica brasileira, cuja população experimenta o envelhecimento populacional ^26^^,^^27^. Estudos apontam o envelhecimento da população carcerária como um fenômeno comum em países desenvolvidos ^28^^,^^29^^,^^30^, observado no Brasil em menor escala.

O número de pessoas privadas de liberdade por vaga se manteve estável no período, expondo que a superlotação dos presídios não é um problema recente ^25^. Talvez a taxa de ocupação não pareça alarmante à primeira vista. Contudo, como bem expõem Winter & Garrido ^31^, imaginando-se 17 pessoas em uma cela que só cabem 10, pode-se visualizar a seriedade da condição de habitação dos presídios brasileiros. Ainda, salienta-se que existem diferenças entre os estados ^32^.

As prevalências de TB aqui apresentadas refletem a literatura vigente, sempre muito superiores nas pessoas privadas de liberdade ^33^^,^^34^. Enquanto para a população brasileira a principal tendência é de queda nas taxas, para a população carcerária, apenas mulheres e idosos apresentam tendência de queda. Ao se analisar o total de custodiados, essa tendência segue no sentido contrário, fato já reconhecido e publicado pelo Ministério da Saúde ^35^. Quanto às razões, com numeradores determinados pelas pessoas privadas de liberdade e suas dimensões muito superiores aos correlatos não prisionais que determinam o denominador, as variações nas razões serão basicamente estabelecidas pela população carcerária.

A queda na tendência de TB em privados de liberdade a partir de 2011 não é concomitante com nenhum grande evento normativo em saúde prisional, além da já citada vigência do PNSSP desde 2003 ^11^. A Política Nacional de Atenção Básica (PNAB), por exemplo, não cita a população carcerária ^36^. Por outro lado, a prestação de contas do DEPEN do ano de 2011 declara a aquisição de equipamentos e melhorias de 42 unidades básicas de saúde (UBS) em estabelecimentos penais, contemplando todas as UF ^37^.

Além disso, os relatórios de gestão do DEPEN, disponíveis até 2015, pouco deixam claro quais investimentos em infraestrutura de saúde foram concluídos de fato com recursos do Fundo Penitenciário Nacional. Chamam a atenção a aquisição de equipamentos de saúde para 7 UF em 2007 ^38^; investimentos em estrutura física e equipamentos de saúde para 4 estados; e repasse de mais de 2,5 milhões para o Ministério da Saúde em 2008 ^39^. Ainda, em 2014, foram guarnecidas 247 UBS nas prisões, com investimento em todos os estados ^40^ e, em 2015, 24 convênios para aparelhamento de 632 UBS ^41^.

Cabe enfatizar que projetos de saúde, sobretudo aqueles direcionados especificamente para TB, podem aumentar a detecção de casos e, consequentemente, a prevalência da doença estudada nos projetos. Assim sendo, podem estar ligados ao aumento da prevalência de TB a partir de 2015 a própria PNAISP de 2014 ^12^; o projeto *TB Reach*^42^^,^^43^, entre 2015 e 2016; e, a partir de 2017, o projeto *Prisões Livres de Tuberculose*^44^.

No projeto *TB Reach* foi enfatizada a detecção de pessoas privadas de liberdade com TB no Brasil ^42^, instituindo, além de rastreamento, atividades educativas ^43^. Já no projeto *Prisões Livres de Tuberculose*, o Ministério da Justiça repassou em 2017 R$ 27,5 milhões à Fundação Oswaldo Cruz (Fiocruz) para promover ações de educação, organização dos fluxos de assistência e ações diversas em saúde ^42^. Assim, antes de se pronunciar sobre um possível insucesso de políticas públicas, deve-se considerar tais programas impactando positivamente a identificação de casos e, consequentemente, aumentando a prevalência de TB entre 2015 a 2019.

A literatura mostra que os homens têm carga maior de TB em comparação às mulheres ^45^. Neste estudo, isso foi verdade para a população geral não privada de liberdade (Figura 1b). Todavia, para população prisional (Figura 1a), isso aconteceu só ao final do período analisado, quando a prevalência de TB em pessoas privadas de liberdade do sexo feminino diminui até tornar-se menor que em pessoas privadas de liberdade de ambos os sexos. Deve-se relatar que, juntamente com a PNAISP, em 2014, foi promulgada a Política Nacional de Atenção às Mulheres em Situação de Privação de Liberdade e Egressas do Sistema Prisional (PNAMPE), ampliando o amparo legislativo às pessoas privadas de liberdade do gênero feminino ^46^.

Quanto à faixa etária, no Brasil, a transição epidemiológica acontece com o aumento das doenças crônicas comuns à senilidade e com reemergência ou presença constante das doenças infecciosas ^47^. Para a TB, no entanto, a partir de 2010, os coeficientes de incidência em pessoas com 60 ou mais anos caem e chegam a se igualar ao de adultos mais jovens ^47^. Para população carcerária do mesmo grupo etário, a prevalência também tem queda contínua, mas sempre muito superior ao total da população prisional e ao do total de idosos do Brasil, semelhante a outros países do mundo ^48^.

Já o percentual de testagem de HIV em pessoas privadas de liberdade com TB teve aumento relevante até 2015, quando atingiu seu máximo de 83,3%, seguido por tênue queda, porém não significativa, ou seja, atingindo relativa estabilidade da série. Isso pode ter acontecido em virtude do efeito teto do indicador. O aumento da testagem para diagnóstico da coinfecção já foi apresentado em outros contextos brasileiros ^47^^,^^49^^,^^50^. Além disso, uma das estratégias do Plano Nacional pelo Fim da Tuberculose como problema de saúde pública era oferecer testagem para HIV a todas as pessoas com TB ^51^.

Vale ressaltar que a TB no sistema prisional enfrenta entraves tanto intramuros, como dificuldade de utilização de laboratórios próprios do sistema prisional ^18^, quanto extramuros, desde conexão entre a saúde prisional e outros pontos da Rede de Atenção à Saúde (RAS) ^52^ até a notificação compulsória e sua baixa completude de dados relacionados à população penitenciária ^19^.

Os resultados deste estudo devem ser interpretados levando-se em consideração suas limitações, entre elas a imperiosa necessidade de se adaptar à forma e qualidade dos dados secundários disponíveis, além de assumir a homocedasticidade das médias estimadas pelo modelo incluídas no modelo de regressão Joinpoint. Outra barreira é o fato de a TB ser a única doença que indica a população carcerária no SINAN, portanto, a única doença marcadora da situação de saúde prisional. A alteração do campo de “presídio” para “pessoas privadas de liberdade” na ficha de notificação em 2014 dificulta o estabelecimento de séries temporais. A única fonte pública de informações sociodemográficas sobre a população prisional é proveniente do DEPEN e não está disponível em formato de banco de dados. Há de se citar ainda o efeito teto da testagem de HIV, que restringe a observação de detalhes ao se aproximar do seu valor máximo.

As grandes fortalezas deste estudo são: sua abrangência nacional; seu foco em pessoas mais vulneráveis (mulheres e idosos) entre os tão vulneráveis; sua metodologia robusta e adequada para o objetivo; pareamento de mudanças de tendência nos desfechos com políticas de saúde geral e/ou prisional; permitir ainda análise de possíveis períodos de implementação de tais políticas, principalmente frente à linha temporal que ultrapassa 5 anos da implementação da PNAISP, a mais específica política de saúde para pessoas privadas de liberdade.

## Conclusão

A TB é doença de alta prevalência nos cárceres. Neste estudo, não houve coincidência temporal nas tendências de TB nas populações analisadas, o que sugere que as políticas nacionais de combate à TB não têm o mesmo efeito dentro das prisões. Mesmo a PNAISP mostrou efeitos restritos diante da situação de saúde aqui analisada. Há de se salientar que a concretização dessas políticas não foi objetivo deste artigo. Mesmo assim, alcançou-se resultados relevantes, incluindo comparações que podem impactar decisões e ações de saúde. Além disso, fica colocada a necessidade novos estudos e um aprofundamento sobre questões relevantes à saúde prisional. E dessa forma, colocar em debate possíveis fragilidades e iniquidades de um sistema de saúde que se propõe ser único e universal a todos os brasileiras mas, entretanto, não alcança da mesma forma os custodiados pelo Estado no sistema prisional.
